# *Vital Signs*: Drowning Death Rates, Self-Reported Swimming Skill, Swimming Lesson Participation, and Recreational Water Exposure — United States, 2019–2023

**DOI:** 10.15585/mmwr.mm7320e1

**Published:** 2024-05-23

**Authors:** Tessa Clemens, Briana Moreland, Karin A. Mack, Karen Thomas, Gwen Bergen, Robin Lee

**Affiliations:** 1 Division of Injury Prevention, National Center for Injury Prevention and Control, CDC.

## Abstract

**Introduction:**

Drowning is the cause of approximately 4,000 U.S. deaths each year and disproportionately affects some age, racial, and ethnic groups. Infrastructure disruptions during the COVID-19 pandemic, including limited access to supervised swimming settings, might have affected drowning rates and risk. Data on factors that contribute to drowning risk are limited. To assess the potential impact of the pandemic on drowning death rates, pre– and post–COVID-19 pandemic rates were compared.

**Methods:**

National Vital Statistics System data were used to compare unintentional drowning death rates in 2019 (pre–COVID-19 pandemic onset) with those in 2020, 2021, and 2022 (post-pandemic onset) by age, sex, and race and ethnicity. National probability-based online panel survey (National Center for Health Statistics Rapid Surveys System) data from October–November 2023 were used to describe adults’ self-reported swimming skill, swimming lesson participation, and exposure to recreational water.

**Results:**

Unintentional drowning death rates were significantly higher during 2020, 2021, and 2022 compared with those in 2019. In all years, rates were highest among children aged 1–4 years; significant increases occurred in most age groups. The highest drowning rates were among non-Hispanic American Indian or Alaska Native and non-Hispanic Black or African American persons. Approximately one half (54.7%) of U.S. adults reported never having taken a swimming lesson. Swimming skill and swimming lesson participation differed by age, sex, and race and ethnicity.

**Conclusions and Implications for Public Health Practice:**

Recent increases in drowning rates, including those among populations already at high risk, have increased the urgency of implementing prevention strategies. Basic swimming and water safety skills training can reduce the risk for drowning. Addressing social and structural barriers that limit access to this training might reduce drowning deaths and inequities. The U.S. National Water Safety Action Plan provides recommendations and tools for communities and organizations to enhance basic swimming and water safety skills training.

**Summary**

**What is already known about this topic?**

Approximately 4,000 unintentional drowning deaths occur annually in the United States, and demographic disparities exist.

**What is added by this report?**

Compared with unintentional drowning death rates in 2019 (pre–COVID-19 pandemic), rates were significantly higher during 2020, 2021, and 2022, with highest rates among children aged 1–4 years, non-Hispanic American Indian and Alaska Native persons, and non-Hispanic Black or African American persons. National survey data revealed that 55% of U.S. adults have never taken a swimming lesson, and swimming lesson participation differed by demographic characteristics.

**What are the implications for public health practice?**

The U.S. National Water Safety Action Plan provides recommendations for drowning prevention actions, including increasing access to basic swimming and water safety skills training for all persons, which could reduce disparities in unintentional drowning deaths.

## Introduction

Approximately 4,000 persons die from unintentional drowning in the United States each year, and some population groups are disproportionately affected. Drowning is the leading cause of death among children aged 1–4 years and one of the three leading causes of unintentional injury death among persons aged 5–34 years (*1*); the second highest drowning death rate is among adults aged ≥65 years (*2*). Drowning death rates are consistently highest among males, non-Hispanic Black or African American (Black), and non-Hispanic American Indian or Alaska Native (AI/AN) persons (*3*). After decades of decreasing drowning rates in the United States, drowning rates increased, and racial and ethnic disparities widened after the onset of the COVID-19 pandemic (*4*). During the COVID-19 pandemic, persons spent more recreational time in or near water, and availability of supervised swimming settings was limited (*4*).

Basic swimming and water safety skills training is an effective drowning prevention strategy (*5*,*6*); however, some groups, including those who have higher rates of drowning (e.g., AI/AN and Black persons) might have limited access to swimming lessons (*7*). In addition to disparities in access, the availability of swimming lessons was affected by infrastructure disruptions during the COVID-19 pandemic and subsequent lifeguard shortages (*8*). This report describes changes in unintentional drowning death rates by sex, age, and race and ethnicity coinciding with the COVID-19 pandemic and presents national estimates of adults’ self-reported swimming skill, swimming lesson participation, and exposure to recreational water.

## Methods

### Mortality Data Analysis

Unintentional drowning deaths from the National Vital Statistics System (NVSS) mortality files for 2019–2022 were identified using the *International Classification of Diseases, Tenth Revision* underlying cause of death codes W65–W74, V90, and V92.* For each year, drowning death rates (unintentional drowning deaths per 100,000 population) were calculated using U.S. Census Bureau postcensal single race estimates of the residential population as of July 1. Rates (other than age-specific rates) were age-adjusted to the year 2000 U.S. Census Bureau standard population.^†^ Percentage change and corresponding 95% CIs were calculated to compare rates from 2020, 2021, and 2022 with those in 2019, overall and by age group, sex, and race and ethnicity. For example, percentage change from 2019 to 2020 was calculated as ([2020 rate − 2019 rate] / [2019 rate]) × 100. Rates were rounded to four decimal places for all calculations and are presented rounded to one decimal place in this report. Persons of Hispanic or Latino (Hispanic) origin might be of any race but are categorized as Hispanic; all racial groups are non-Hispanic. 

### Survey Data Analysis

Data on adults’ self-reported swimming skill, swimming lesson participation, and water exposure were obtained from Round 2 of the National Center for Health Statistics Rapid Surveys System (RSS),^§^ fielded during October–November 2023. The RSS platform is designed to approximate national representation of the adult U.S. population and collects self-reported health data using two commercially available, statistically sampled national probability-based online panels: 1) National Opinion Research Center, at the University of Chicago’s AmeriSpeak Panel (https://amerispeak.norc.org/) and 2) Ipsos’s KnowledgePanel (https://www.ipsos.com/en-us/solutions/public-affairs/knowledgepanel). Nationally representative weights calibrated to the National Health Interview Survey were created to reduce coverage and nonresponse biases. Variances were estimated using the Taylor series linearization method that takes survey design into account. Self-reported swimming skill, swimming lesson participation, and recreational water exposure were described overall and by sex, age group, and race and ethnicity. Race and ethnicity were categorized as Black, non-Hispanic White (White), Hispanic, and non-Hispanic other race (other). Differences with nonoverlapping 95% CIs were considered significant. This activity was reviewed by CDC, deemed not research, and was conducted consistent with applicable federal law and CDC policy.^¶^

## Results

### Drowning Death Rates

Compared with an overall unintentional drowning death rate of 1.2 per 100,000 persons in 2019, rates were significantly higher in 2020 (1.4; 10.5% increase), 2021 (1.4; 13.7%), and 2022 (1.3; 9.1%) (Table 1). Drowning death rates were higher among males (range = 1.9–2.1) than among females (range = 0.6–0.7) in all years. Compared with rates in 2019, drowning death rates among males were significantly higher in 2020 (2.1; 12.8%), 2021 (2.1; 12.8%), and 2022 (2.0; 6.5%), and rates among females were significantly higher in 2021 (0.7; 22.2%) and 2022 (0.6; 13.7%). Drowning death rates were highest among children aged 1–4 years in all years and increased significantly in 2021 (3.1; 28.9%) and 2022 (3.1; 28.3%) compared with 2019. The next highest death rate occurred among adults aged ≥65 years and, compared with rates in 2019, rates were significantly higher among persons aged 65–74 years in 2022 (1.8; 19.1%) and among persons aged ≥85 years in 2021 (2.4; 49.8%). The largest increase in drowning death rates in 2020 compared with 2019 occurred among persons aged 15–24 (1.4; 31.3%) and 25–34 years (1.3; 21.1%). When examined by race and ethnicity, the highest drowning rates were among AI/AN (range = 2.6–3.1) and Black persons (range = 1.5–1.9) in all years. The largest increases in drowning rates relative to 2019 occurred among Black persons in 2020 (1.8; 22.2%) and 2021 (1.9; 28.3%) and among Hispanic persons in 2022 (1.2; 24.8%).

**TABLE 1:** Changes in drowning* death rates,^†^ by sex, age group, and race and ethnicity — National Vital Statistics System, United States, 2019–2022

Characteristic	2019	2020		2021		2022	
	No. (rate)^§^	No. (rate)^§^	% Change^¶^ (95% CI)	No. (rate)^§^	% Change^¶ ^(95% CI)	No. (rate)^§^	% Change^¶ ^(95% CI)
**Sex**							
Female	953 (0.6)	1,040 (0.6)	9.6 (−0.3 to 19.6)	1,137 (0.7)	22.2 (11.3 to 33.0)	1,113 (0.6)	13.7 (3.5 to 23.9)
Male	3,114 (1.9)	3,549 (2.1)	12.8 (7.2 to 18.3)	3,540 (2.1)	12.8 (7.2 to 18.3)	3,396 (2.0)	6.5 (1.2 to 11.7)
**Age group, yrs**							
<1	34 (0.9)	34 (0.9)	1.3 (−46.9 to 49.4)	38 (1.1)	18.6 (−36.2 to 73.5)	39 (1.1)	17.8 (−36.4 to 72.0)
1–4	382 (2.4)	425 (2.7)	12.9 (−2.7 to 28.5)	476 (3.1)	28.9 (11.6 to 46.3)	461 (3.1)	28.3 (10.9 to 45.7)
5–14	240 (0.6)	208 (0.5)	−13.3 (−29.4 to 2.8)	244 (0.6)	−0.1 (−17.9 to 17.7)	230 (0.6)	−3.9 (−21.3 to 13.5)
15–24	453 (1.1)	593 (1.4)	31.3 (15.2 to 47.4)	475 (1.1)	3.9 (−9.5 to 17.3)	488 (1.1)	3.7 (−9.6 to 17.0)
25–34	503 (1.1)	611 (1.3)	21.1 (6.8 to 35.4)	605 (1.3)	21.5 (7.1 to 35.8)	547 (1.2)	9.8 (−3.5 to 23.1)
35–44	481 (1.2)	568 (1.3)	16.8 (2.6 to 30.9)	642 (1.5)	28.1 (13.0 to 43.2)	549 (1.3)	8.8 (−4.5 to 22.1)
45–54	528 (1.3)	569 (1.4)	9.1 (−3.8 to 22.1)	511 (1.3)	−2.8 (−14.6 to 9.1)	483 (1.2)	−7.5 (−18.9 to 3.9)
55–64	568 (1.3)	639 (1.5)	12.6 (−0.1 to 25.3)	656 (1.5)	14.5 (1.7 to 27.4)	625 (1.5)	11.0 (−1.6 to 23.6)
65–74	475 (1.5)	503 (1.5)	2.4 (−10.4 to 15.3)	545 (1.6)	7.3 (−5.9 to 20.5)	607 (1.8)	19.1 (4.8 to 33.4)
75–84	293 (1.8)	307 (1.9)	1.7 (−14.6 to 18.0)	340 (2.1)	14.4 (−3.5 to 32.2)	342 (2.0)	6.4 (−10.2 to 23.0)
≥85	107 (1.6)	131 (2.0)	21.4 (−9.6 to 52.5)	145 (2.4)	49.8 (12.4 to 87.2)	136 (2.1)	29.4 (−3.3 to 62.2)
Race and ethnicity**							
AI/AN	74 (3.1)	72 (2.9)	−5.1 (−36.4 to 26.3)	62 (2.6)	−14.5 (−43.9 to 14.9)	68 (2.8)	−8.4 (−39.2 to 22.3)
Asian	185 (1.0)	209 (1.1)	8.3 (−13.5 to 30.0)	228 (1.2)	20.2 (−3.4 to 43.9)	183 (0.9)	−8.6 (−27.6 to 10.3)
Black or African American	601 (1.5)	750 (1.8)	22.2 (8.9 to 35.5)	780 (1.9)	28.3 (14.5 to 42.1)	675 (1.6)	9.5 (−2.7 to 21.6)
NH/OPI	—^††^	23 (3.6)	NA	17 (—)	NA	17 (—)	NA
White	2,548 (1.2)	2,791 (1.4)	13.2 (6.8 to 19.7)	2,843 (1.4)	12.5 (6.1 to 18.9)	2,725 (1.3)	4.7 (−1.3 to 10.8)
Hispanic or Latino	573 (0.9)	670 (1.1)	14.9 (1.7 to 28.2)	667 (1.1)	14.9 (1.7 to 28.1)	742 (1.2)	24.8 (10.7 to 38.8)
Multiracial	63 (0.9)	65 (0.9)	1.5 (−38.9 to 41.9)	70 (0.9)	−7.5 (−43.5 to 28.6)	85 (1.0)	9.1 (−31.3 to 49.6)
**Total^§§^**	**4,067 (1.2)**	**4,589 (1.4)**	**10.5 (5.7 to 15.3)**	**4,677 (1.4)**	**13.7 (8.8 to 18.6)**	**4,509 (1.3)**	**9.1 (4.4 to 13.9)**

**Abbreviations:** AI/AN = American Indian or Alaska Native; NA = not applicable; NH/OPI = Native Hawaiian or other Pacific Islander.

* *International Classification of Diseases, Tenth Revision* underlying cause of death codes W65–W74, V90, and V92.

^†^ Unintentional drowning deaths per 100,000 population.

^§^ All rates are age-adjusted to the 2000 U.S. Census Bureau population using the direct method, except the age-group specific rates.

^¶^ Percentage change was compared with 2019 and was calculated comparing rates from each year to 2019. For example, percentage change from 2019 to 2020 was calculated as ([2020 rate − 2019 rate] / [2019 rate]) × 100; rates were rounded to four decimal places for all calculations and rounded to one decimal place in the table. Percentage change is considered significant if the 95% CI excludes zero.

** Hispanic or Latino ethnicity includes persons of any race. Racial groups exclude persons of Hispanic ethnicity. Persons with unknown ethnicity are excluded from race and ethnicity groups but are included in the overall total.

^††^ Dash indicates death counts based on fewer than 10 deaths suppressed for confidentiality; death rates based on fewer than 20 deaths are suppressed because of unreliability.

**^§§^** Total rates include race and ethnicity and age group not stated.

### Swimming Skill and Swimming Lessons

An estimated 40 million adults (15.4% of survey respondents) reported not knowing how to swim (Table 2). More than one half of adults (54.7%) reported never having taken a swimming lesson. Self-reported swimming skill and swimming lesson participation differed by sex, age, and race and ethnicity. Women were significantly more likely than were men to report that they did not know how to swim (19.4% versus 11.2%), and adults aged ≥65 years were more likely to report not knowing how to swim (18.6%) than were those aged 18–29 years (12.4%). Approximately one third (36.8%) of Black adults reported not knowing how to swim, a significantly higher percentage than that of Hispanic adults (25.8%), White adults (6.9%), and adults of other racial or ethnic groups (22.4%). Compared with White adults, approximately one half (51.8%) of whom reported ever having taken a swimming lesson, a significantly lower percentage of Black adults (36.9%) and Hispanic adults (28.1%) had ever taken a swimming lesson.

**TABLE 2:** Self-reported swimming skill, swimming lesson participation, and exposure to recreational water during the previous 6 months among adults, by sex, age group, and race and ethnicity — National Center for Health Statistics Rapid Surveys System, United States, October–November 2023

Characteristic	Total	Sex		Age group, yrs				Race and ethnicity*			
		Female	Male	18–29	30–44	45–64	≥65	Black or African American	White	Hispanic or Latino	Other
**Swim skill level^†^**											
**Does not know how to swim**											
Weighted % (95% CI)	**15.4 (14.3–16.6)**	19.4 (17.8–21.2)	11.2 (9.8–12.7)	12.4 (9.9–15.3)	15.6 (13.3–18.1)	15.0 (13.1–16.9)	18.6 (16.6–20.7)	36.8 (32.4–41.4)	6.9 (6.1–7.8)	25.8 (22.5–29.3)	22.4 (17.9–27.5)
Weighted no. (× 1,000)	**39,527**	25,529	13,998	6,848	10,299	11,649	10,732	11,719	10,896	11,429	4,545
**Comfortable in water where they can stand**											
Weighted % (95% CI)	**17.0 (15.9–18.1)**	19.4 (17.8–21.0)	14.4 (12.9–16.0)	19.8 (16.5–23.5)	14.5 (12.6–16.6)	14.8 (13.1–16.6)	20.0 (18.0–22.1)	27.0 (23.0–31.2)	13.7 (12.6–14.9)	20.0 (16.9–23.3)	20.4 (16.3–25.0)
Weighted no. (× 1,000)	**43,547**	25,473	18,075	10,923	9,574	11,529	11,521	8,591	21,690	8,836	4,128
**Can swim in water over their head**											
Weighted % (95% CI)	**31.8 (30.4–33.2)**	30.8 (29.0–32.7)	32.8 (30.8–34.9)	32.9 (29.4–36.7)	30.0 (27.6–32.6)	32.1 (29.8–34.4)	32.4 (30.0–34.9)	18.8 (15.5–22.4)	36.7 (35.0–38.5)	25.7 (22.3–29.3)	29.1 (24.3–34.3)
Weighted no. (× 1,000)	**81,656**	40,506	41,150	18,147	19,810	24,969	18,730	5,977	57,977	11,371	5,898
**Can swim multiple strokes efficiently**											
Weighted % (95% CI)	**35.8 (34.5–37.2)**	30.4 (28.6–32.2)	41.6 (39.5–43.7)	34.8 (31.2–38.6)	39.9 (37.0–42.8)	38.2 (35.9–40.5)	29.0 (26.8–31.4)	17.4 (14.2–21.0)	42.6 (40.9–44.4)	28.5 (25.1–32.1)	28.1 (23.5–33.1)
Weighted no. (× 1,000)	**91,991**	39,897	52,095	19,170	26,314	29,745	16,762	5,549	67,299	12,626	5,694
**Swimming lessons^§^**											
**Has taken private swimming lesson from a professional^§^**											
Weighted % (95% CI)	**14.2 (13.2–15.2)**	13.5 (12.2–14.9)	14.9 (13.4–16.4)	16.2 (13.5–19.2)	15.0 (13.0–17.2)	13.5 (12.0–15.1)	12.2 (10.6–13.9)	12.6 (9.8–15.8)	15.7 (14.4–16.9)	10.0 (7.8–12.7)	14.3 (10.9–18.4)
Weighted no. (× 1,000)	**36,320**	17,756	18,564	8,899	9,897	10,462	7,063	4,024	24,651	4,429	2,903
**Has taken group swimming lesson from a professional^§^**											
Weighted % (95% CI)	**33.9 (32.5–35.3)**	34.1 (32.3–35.9)	33.7 (31.6–35.8)	37.0 (33.2–40.9)	35.5 (32.8–38.4)	32.7 (30.4–35.0)	30.7 (28.4–33.0)	25.1 (21.4–29.1)	39.7 (38.0–41.4)	19.6 (16.6–22.9)	34.6 (29.7–39.8)
Weighted no. (× 1,000)	**87,008**	44,809	42,199	20,392	23,430	25,431	17,756	8,002	62,623	8,709	7,015
**Has taken other swimming lesson^§^**											
Weighted % (95% CI)	**7.7 (7.0–8.5)**	7.2 (6.3–8.2)	8.2 (7.2–9.4)	5.4 (3.9–7.2)	7.7 (6.2–9.3)	8.4 (7.2–9.7)	9.1 (7.7–10.6)	8.1 (6.0–10.5)	8.3 (7.4–9.3)	5.3 (3.9–7.1)	7.9 (5.3–11.4)
Weighted no. (× 1,000)	**19,799**	9,471	10,328	2,977	5,057	6,539	5,225	2,579	13,091	2,352	1,609
**Has ever taken a swimming lesson^§^**											
Weighted % (95% CI)	**45.3 (43.8–46.8)**	44.8 (42.9–46.8)	45.8 (43.6–48.0)	46.9 (42.9–50.9)	47.0 (44.0–50.1)	44.7 (42.3–47.1)	42.6 (40.1–45.1)	36.9 (32.7–41.2)	51.8 (50.1–53.5)	28.1 (24.7–31.6)	46.6 (41.2–52.1)
Weighted no. (× 1,000)	**115,996**	58,772	57,224	25,779	30,925	34,727	24,564	11,704	81,609	12,356	9,423
**Time at pool^¶^**											
**None**											
Weighted % (95% CI)	**48.8 (47.4–50.3)**	47.5 (45.5–49.5)	50.3 (48.2–52.3)	41.0 (37.1–44.9)	41.6 (38.8–44.6)	50.6 (48.2–53.0)	62.1 (59.6–64.5)	67.0 (62.7–71.1)	43.9 (42.2–45.6)	47.7 (43.9–51.6)	58.9 (53.4–64.3)
Weighted no. (× 1,000)	**125,352**	62,466	62,886	22,564	27,368	39,494	35,926	21,451	69,364	21,012	11,937
**1–6 days**											
Weighted % (95% CI)	**28.2 (26.9–29.5)**	28.8 (27.0–30.7)	27.5 (25.6–29.3)	36.5 (32.9–40.3)	31.6 (28.9–34.3)	26.0 (23.9–28.2)	19.2 (17.3–21.3)	22.5 (18.8–26.5)	28.5 (26.9–30.1)	32.9 (29.2–36.7)	25.8 (21.0–31.1)
Weighted no. (× 1,000)	**72,253**	37,890	34,363	20,108	20,744	20,280	11,121	7,202	44,999	14,473	5,229
**≥7 days**											
Weighted % (95% CI)	**23.0**	23.7	22.3	22.5	26.8	23.4	18.7	10.5	27.6	19.4	15.2
	**(21.8–24.2)**	(22.1–25.3)	(20.6–24.0)	(19.3–25.9)	(24.3–29.4)	(21.4–25.5)	(16.7–20.7)	(8.0–13.5)	(26.1–29.2)	(16.6–22.6)	(11.8–19.2)
Weighted no. (× 1,000)	**59,050**	31,158	27,893	12,380	17,613	18,251	10,807	3,377	43,573	8,555	3,087
Time at other body of water**											
**None**											
Weighted % (95% CI)	**55.4 (54.0–56.9)**	55.5 (53.6–57.5)	55.3 (53.2–57.4)	50.5 (46.5–54.5)	48.6 (45.6–51.6)	54.7 (52.3–57.2)	69.0 (66.5–71.3)	74.0 (70.0–77.7)	49.8 (48.1–51.6)	58.6 (54.5–62.7)	61.7 (56.2–66.9)
Weighted no. (× 1,000)	**141,865**	72,676	69,189	27,694	31,970	42,484	39,717	23,671	78,419	25,807	12,454
**1–6 days**											
Weighted % (95% CI)	**27.5 (26.2–28.9)**	28.3 (26.5–30.2)	26.7 (24.9–28.7)	33.8 (30.2–37.6)	31.1 (28.4–33.8)	26.4 (24.4–28.6)	19.0 (17.1–21.2)	20.3 (16.9–24.1)	29.3 (27.7–31.0)	28.0 (24.5–31.7)	24.6 (20.1–29.6)
Weighted no. (× 1,000)	**70,489**	37,058	33,431	18,560	20,438	20,521	10,970	6,503	46,143	12,326	4,974
**≥7 days**											
Weighted % (95% CI)	**17.0**	16.1	17.9	15.7	20.3	18.8	12.0	5.6	20.8	13.3	13.7
	**(16.0–18.1)**	(14.7–17.6)	(16.4–19.5)	(13.0–18.8)	(18.1–22.7)	(16.9–20.8)	(10.4–13.7)	(3.8–8.0)	(19.4–22.3)	(10.7–16.4)	(10.2–17.9)
Weighted no. (× 1,000)	**43,519**	21,098	22,422	8,636	13,371	14,606	6,906	1,805	32,776	5,872	2,766

**Source:** National Center for Health Statistics (NCHS), Rapid Surveys System, Round 2, October–November 2023. All estimates shown meet the NCHS standards of reliability. https://www.cdc.gov/nchs/rss/rapid-surveys-system.html

* Persons identified as Hispanic or Latino (Hispanic) might be of any race. Persons identified as Black or African American, White, or Other are all non-Hispanic. Other race includes persons who identify as Asian, American Indian or Alaska Native, Middle Eastern or North African, Native Hawaiian or other Pacific Islander, or multiracial.

^†^ Respondents were asked, “How would you rate your swimming skill level?”

^§^ Respondents were asked, “Have you taken private swim lessons from a professional or certified instructor?” and “Have you taken group swim lessons from a professional or certified instructor?” Respondents who answered “no” (or did not respond to the question) were asked, “Have you ever taken a swim lesson?” (other swim lesson). Respondents were coded as “Has ever taken a swim lesson” if they responded “yes” to any of those three questions.

^¶^ Respondents were asked, “In the past 6 months, on how many days in total did you spend time in or around a swimming pool?”

** Respondents were asked, “In the past 6 months, on how many days in total did you go swimming, boating, fishing, or participate in water sports in another body of water such as an ocean, lake, river, or stream?”

### Recreational Water Exposure

Approximately one half (51.2%) of adults reported spending time at a swimming pool in the preceding 6 months, and 44.5% reported spending time near other bodies of water, such as an ocean, lake, or river. Reported prevalence of having spent time at a swimming pool declined with increasing age: 59.0% of adults aged 18–29 and 58.4% of those aged 30–44 years reported having spent time at a swimming pool, compared with 37.9% of adults aged ≥65 years. Similarly, 49.5% of adults aged 18–29, 51.4% of those aged 30–44, and 45.3% of adults aged 45–64 years reported spending one or more days at other bodies of water, compared with 31.0% of older adults. A significantly higher percentage of Black adults reported spending no time at a swimming pool (67.0%) or other body of water (74.0%) during the previous 6 months than did White adults (43.9% and 49.8%, respectively) or Hispanic adults (47.7% and 58.6%, respectively).

## Discussion

Unintentional drowning death rates in the United States were higher in 2020, 2021, and 2022 than in 2019. The increases in drowning deaths coinciding with the COVID-19 pandemic and new national estimates of self-reported swimming skill, swimming lesson participation, and recreational water exposure highlight the need to increase access to effective drowning prevention strategies such as basic swimming and water safety skills training to reduce drowning risk.

This analysis identified notable increases in drowning death rates among groups that were already disproportionately affected, including children aged 1–4 years, older adults, and Black persons. Although drowning rates among AI/AN persons did not increase, rates in this group continued to be the highest among any racial or ethnic group. Drowning death rates have not historically been disproportionately high among Hispanic persons; however, drowning death rates among Hispanic persons were significantly higher in 2020, 2021, and 2022, compared with the rate in 2019. Findings related to adults’ exposure to recreational water suggest that population-based drowning rates might be underestimating disparities. For example, older adults and Black adults reported significantly less exposure to recreational water than did other adults, indicating that if drowning rates were calculated based on exposure rather than population, rates in these groups would be even higher.

Increasing unintentional drowning deaths among children aged 1–4 years might partly reflect disruptions caused by the COVID-19 pandemic. Although children spent more time at home, where exposure to backyard pools and other water sources might have increased, and family routines were modified, drowning death rates among children aged 1–4 years did not increase significantly in 2020, when these conditions were most likely to prevail. Significant increases in drowning death rates in this age group during 2021 and 2022 underscore the importance of implementing effective drowning prevention strategies including installing four-sided pool fencing; providing close, constant, and attentive supervision; using life jackets; and beginning swimming lessons as soon as children are developmentally ready (*9*).

Unintentional drowning death rates among persons aged 15–44 years increased in 2020. Although information on alcohol use was not included in this analysis, previous research has indicated that alcohol use is a major risk factor for drowning among teens and adults: a recent meta-analysis found that 31% of drowning deaths were attributable to alcohol (*10*). Previous studies have identified high self-reported alcohol use during aquatic activities, with 25%–61% of persons aged 15–34 years reporting alcohol use around water (*11*). Drowning prevention strategies for teens and adults should include comprehensive approaches to reducing alcohol use around water, in addition to learning basic swimming and water safety skills and wearing life jackets.

Unintentional drowning death rates among persons aged 65–74 years increased in 2022 and among persons aged ≥85 years in 2021. These increases align with recent trends: drowning death rates among adults aged ≥65 years have been increasing for decades (*2*)*, *and survey respondents in this age group were significantly less likely to report knowing how to swim than were young adults*.* More work is needed to understand the circumstances of drowning among older adults in the United States and to develop tailored drowning prevention strategies.

Survey data revealed lower self-reported swimming skill and swimming lesson participation among some of the groups with the highest drowning death rates or the highest percentage increases in drowning death rates, including among Black and Hispanic persons and older adults. For example, approximately one third of Black adults reported not knowing how to swim, and significantly more Black and Hispanic adults than White adults reported never having taken a swimming lesson. Taking formal swimming lessons reduces the risk for drowning (*5*,*6*). Disparities in access to swimming skills training might be one factor contributing to disproportionate drowning death rates among some groups. These disparities are influenced by complex historical, structural, and social factors. Research suggests that differences in participation in swimming has been affected by inequitable structural environments (e.g., availability of swimming pools) and social exclusivity (*7*). Historically, racially segregated pools led to fewer swimming options for Black persons, and available pools were often too small and shallow for swimming (*12*). When integration of public pools was mandated, many pools closed, fewer new pools were built, and private swimming clubs emerged that restricted access for Black persons through discriminatory membership or residential requirements (*12*).

Barriers to swimming participation persist: a recent survey identified nearby swimming pool access, among other social and structural factors, as a major barrier to swimming skills training reported by Black and AI/AN persons (*13*), two racial groups at increased risk for drowning. The COVID-19 pandemic also affected the availability of swimming lessons because local restrictions caused many pools to close, and once they reopened, they faced staffing shortages (*8*). Examining the factors that contribute to inequities in learning to swim is important for developing and implementing strategies that increase access to culturally responsive basic swimming and water safety skills training programs. The U.S. National Water Safety Action Plan** (2023–2032) serves as a roadmap for reducing drowning and provides a framework for communities to use to develop and implement local action plans. The plan calls upon communities and organizations to build or revitalize publicly accessible swimming pools; provide affordable, accessible, and culturally competent swimming lessons; embed diversity, equity, inclusion, and cultural training into aquatics programs; and hire and train diverse personnel. The plan includes tools to support communities in implementing these and other actions to increase access to swimming lessons and thereby reduce the risk for drowning (*14*).

### Limitations

The findings in this report are subject to at least five limitations. First, racial and ethnic group designation might involve misclassification that could lead to over- or underestimating the rates among some groups (*15*). Second, the 2021 and 2022 NVSS population estimates are based on the blended base estimates calculated by the U.S. Census Bureau, which differ from the method for calculating population estimates in previous years (*16*,*17*). This change is specifically noticeable in the population aged ≥85 years in 2021 and might partially contribute to the large increase in unintentional drowning death rates in that population. Third, this analysis did not include information on the circumstances of the drowning deaths, which could guide the development of tailored drowning prevention strategies. Fourth, the RSS web-based panel survey has a lower response rate than do other large-scale national surveys conducted by CDC and might underrepresent certain subpopulations, increasing the potential for nonresponse bias (*18*). RSS reduces nonresponse bias through calibration and weighting of RSS data to benchmark National Center for Health Statistics surveys that use methods to maximize relevance, accuracy, and reliability but require a longer period for data collection and processing than do rapid surveys (*19*). Finally, respondents in both panels complete demographic questions before participating in any surveys; therefore, demographic measures were not collected at the same time as the swimming skill and water exposure measures, and this process might lead to some misclassification.

### Implications for Public Health Practice

Increases in unintentional drowning death rates during 2020–2022, including increases among populations that were already at elevated risk, such as young children, older adults, and Black persons, have highlighted the urgency of implementing evidence-based prevention strategies that can have immediate and lasting benefits. Basic swimming and water safety skills training can reduce the risk for drowning (*5*,*6*), but social and structural barriers to accessing this training persist; these barriers disproportionately affect groups at the highest risk for drowning. Addressing system-level barriers to accessing basic swimming and water safety skills training could curb increasing drowning rates and reduce inequities.

## Footnotes

* W65–W74 = accidental drowning and submersion; V90 = accident to watercraft causing drowning and submersion; and V92 = drowning and submersion due to accident on board watercraft, without accident to watercraft.

†
https://wonder.cdc.gov/wonder/help/ucd-expanded.html#Age-Adjusted%20Rates

§
https://www.cdc.gov/nchs/rss/rapid-surveys-system.html

¶ 45 C.F.R. part 46, 21 C.F.R. part 56; 42 U.S.C. Sect. 241(d); 5 U.S.C. Sect. 552a; 44 U.S.C. Sect. 3501 et seq.

**
https://www.watersafetyusa.org/nwsap.html
